# A change in the study evaluation paradigm reveals that larynx preservation compromises survival in T4 laryngeal cancer patients

**DOI:** 10.1186/s12885-017-3608-7

**Published:** 2017-09-01

**Authors:** Gerhard Dyckhoff, Peter K. Plinkert, Heribert Ramroth

**Affiliations:** 1 Department of Otorhinolaryngology, Head and Neck Surgery, University of Heidelberg, Im Neuenheimer Feld 400, 69120 Heidelberg, Germany; 2 Institute of Public Health, University of Heidelberg, INF 324, 69120 Heidelberg, Germany

**Keywords:** Laryngeal cancer, Advanced stage, Larynx preservation, Laryngectomy, Outcome

## Abstract

**Background:**

Larynx preservation (LP) is recommended for up to low-volume T4 laryngeal cancer as an evidence-based treatment option that does not compromise survival. However, a reevaluation of the current literature raises questions regarding whether there is indeed reliable evidence to support larynx preservation for T4 tumor patients.

**Methods:**

In an observational cohort study of 810 laryngeal cancer patients, we evaluated the outcomes of all T4 tumor patients treated with primary chemo-radiotherapy (CRT) or primary radiotherapy alone (RT) compared with upfront total laryngectomy followed by adjuvant (chemo)radiotherapy (TL + a[C]RT). Additionally, we reevaluated the studies that form the evidence base for the recommendation of LP for patients with up to T4 tumors (Pfister et al., J Clin Oncol 24:3693–704, 2006).

**Results:**

The evaluation of all 288 stage III and IV patients together did not show a significant difference in overall survival (OS) between CRT-LP and TL + a(C)RT (hazard ratio (HR) 1.23; 95% confidence interval (CI): 0.82–1.86; *p* = 0.31) using a multivariate proportional hazard model. However, a subgroup analysis of T4 tumor patients alone (*N* = 107; 13.9%) revealed significantly worse OS after CRT compared with TL + a(C)RT (HR 2.0; 95% CI: 1.04–3.7; *p* = 0.0369). A reevaluation of the subgroup of T4 patients in the 5 LP studies that led to the ASCO clinical practice guidelines revealed that only 21–45 T4 patients had differential data on survival outcome. These data, however, showed a markedly worse outcome for T4 patients after LP.

**Conclusions:**

T4 laryngeal cancer patients who reject TL as a treatment option should be informed that their chance of organ preservation with primary conservative treatment is likely to result in a significantly worse outcome in terms of OS. Significant loss of survival in T4 patients after LP is also confirmed in recent literature.

## Background

In the landmark larynx preservation (LP) studies [1–3], common practice has been to investigate and evaluate locally advanced stage III and IV cancers of the larynx or hypopharynx together. These groups comprise T4 carcinoma as well as T2 and T3 cancers. The results of these studies led to the American Society of Clinical Oncology (ASCO) 2006 clinical practice guidelines for the use of larynx preservation strategies [4]. These guidelines recommend that “for most patients with T3 or T4 disease without tumor invasion through cartilage into soft tissues, a larynx preservation approach is an appropriate, standard treatment option, and concurrent chemo-radiotherapy is the most widely applicable approach.” [4] Furthermore, they state that with “further surgery reserved for salvage, survival is not compromised.” [4] These guidelines are currently the official standard for avoiding total laryngectomy, particularly in the United States [5], as recent reviews have reconfirmed [6–9]. Thus, in patients with early T4 disease, LP is explicitly recommended. According to the current National Comprehensive Cancer Network (NCCN) treatment guidelines, concurrent chemoradiation should be considered only for “selected T4a patients who decline surgery” [10]. As a result, one might expect that only a minority of carefully selected T4a laryngeal cancer patients are treated using primary conservative treatment. However, nearly two-thirds of patients with T4a disease undergo LP chemo-radiation [11].

We evaluated the outcomes of all T4 laryngeal cancer patients between 1998 and 2004 in a study region covering a population of approximately 2.7 million people with a follow-up of up to 17 years.

Motivated by the poor outcome after LP in this subgroup, we reevaluated the literature cited in the ASCO 2006 guidelines to investigate whether there is indeed reliable evidence of equal survival in T4 laryngeal cancer patients who receive primary chemo-radiotherapy (CRT) or radiation therapy alone (RT) compared with those who undergo upfront total laryngectomy (TL).

Furthermore, we searched the literature for studies published since 2006 providing evidence of the outcomes of T4 laryngeal cancer patients after LP compared with primary surgical treatment.

## Methods

From 1998 to 2004, all laryngeal cancer patients (*N* = 810) treated in the Southwestern region of Germany (covering a population of 2.7 million people) were identified as part of an observational cohort study and followed for at least 10 years. In this region, laryngeal cancer is exclusively treated in the clinics from which the cases were obtained. Local practitioners were also contacted to identify possible cases sent to more distant clinics and to verify complete case ascertainment.

Demographic data and clinical information were extracted from hospital medical records using a standardized form. Vital status and date and cause of death were requested from local registries.

Overall survival (OS) rates were calculated using the Kaplan–Meier method. Regression analysis was performed using multivariate proportional hazards models. The overall survival rates of CRT and RT, both with the option of salvage TL, were compared with those of surgery (i.e., upfront TL in T4 cases) with adjuvant radiotherapy or adjuvant chemo-radiotherapy, as indicated by stage (TL+/-a[C]RT). Survival time was measured as the time from the first diagnosis until death or until 21 March 2015. For the analysis, patients who migrated out of Germany were censored after 1 month of emigration. Only OS estimates are presented. *P*-values below 0.05 were considered statistically significant.

The following variables, which showed an effect in the univariate analysis (*p* < 0.20), were included in the multivariate analysis as explanatory variables: age at first diagnosis (continuous), tumor location, TNM classification, comorbidities, recurrences and second primary carcinomas and therapy approach. Backward selection was used to obtain a final model. Proportional hazards assumption was checked by adding a time-dependent version of all the variables in the model [12]. The assumption was met for all variables. The metastatic status could not be evaluated as M1 status could be clearly determined for only 5 patients. Comorbidity conditions were determined using the Charlson comorbidity index (CCI), which summarizes 18 different comorbidities, weighted by severity, in a single score [13]. For this analysis, we considered the binary form of the variable, which is set to one for CCI values of two or higher. The development of local or regional recurrence or a second primary carcinoma (SPC) was included in the model as a time-dependent covariate. For the date of diagnosis of a recurrence or an SPC, the corresponding variable was set to one. SAS 9.4 statistical software was used for all analyses.

Additionally, the literature quoted in the ASCO 2006 guidelines as the evidence base for recommending LP for patients with up to T4 cancer was reevaluated. According to the classical meaning, LP studies were defined as those that included either advanced-stage laryngeal or hypopharyngeal cancers that require or are amenable to laryngectomy and are treated with LP as an alternative to TL. To the extent that the available data permitted, we checked i.) the number of T4 patients who eventually received primary conservative treatment compared with those who had been assigned to the conservative treatment arm and ii.) the outcomes of this subgroup. A further literature search was conducted to identify the studies that have investigated the treatment of T4 laryngeal patients to date.

## Results

During the seven-year recruitment period, 810 laryngeal cancer patients were identified. For the current analyses, 41 patients were excluded as they either received no treatment with curative intent (*n* = 28) or their tumor stage was unknown (*n* = 13).

The median follow-up time for the remaining 769 patients was 8.3 years, with a range from 14 days to 16.8 years.

A subgroup of 288 patients (37.5%) was classified as advanced stage and received treatment with curative intent. The subgroup included 119 stage III (15.5%) and 169 stage IV (22.0%) patients. Most of those patients were treated with surgery (*n* = 238); 30 (10.4%) were treated with CRT, and 20 (6.9%) were treated with RT alone. Additional information regarding the demographic and clinical characteristics of the three treatment groups is provided in Table 1.

**Table 1:** Demographic and clinical characteristics of the three treatment groups

Charactersitic	Category	OP+/−a(C)RT N (%)	CRT N (%)	RT N (%)
Total		684 (100)	40 (100)	45 (100)
Age (continuous)^a^		61.9 (9.7)	61.2 (11.1)	64.6 (9.8)
Sex	Males	626 (91.5)	33 (82.5)	36 (80.0)
	Females	58 (8.5)	7 (17.5)	9 (20.0)
CCI	0	494 (72.2)	33 (82.5)	22 (48.9)
	1	100 (14.6)	1 (2.5)	15 (33.3)
	2	63 (9.2)	5 (12.5)	6 (13.3)
	3+	27 (3.9)	1 (2.5)	2 (4.4)
Tumour location	glottic	435 (63.6)	8 (20.0)	23 (51.1)
	supraglottic	168 (24.6)	22 (55.0)	14 (31.1)
	subglottic	13 (1.9)	1 (2.5)	1 (2.2)
	transglottic	42 (6.1)	6 (15.0)	3 (6.7)
	unknown	26 (3.8)	3 (7.5)	4 (8.9)
Stage	I	304 (44.4)	3 (7.5)	10 (22.2)
	II	142 (20.8)	7 (17.5)	15 (33.3)
	III	103 (15.1)	10 (25.0)	6 (13.3)
	IV	135 (19.7)	20 (50.0)	14 (31.1)
T stage	1	319 (46.6)	5 (12.5)	12 (26.7)
	2	176 (25.7)	11 (27.5)	18 (40.0)
	3	103 (15.1)	11 (27.5)	7 (15.6)
	4	86 (12.6)	13 (32.5)	8 (17.8)
N stage	0	528 (77.2)	20 (50.0)	30 (66.7)
	1	40 (5.8)	3 (7.5)	4 (8.9)
	2	75 (11.0)	12 (30.0)	8 (17.8)
	3	3 (0.4)	3 (7.5)	2 (4.4)
	unknown	38 (5.6)	2 (5.0)	1 (2.2)
Grading	1	47 (6.9)	1 (2.5)	3 (6.7)
	2	420 (61.4)	16 (40.0)	16 (35.6)
	3,4	118 (17.3)	5 (12.5)	7 (15.6)
	0, x	99 (14.5)	18 (45.0)	19 (42.2)
Laser		452 (66.1)	−	−
Partial resection		59 (8.6)	−	−
TL		173 (25.3)	−	−
RT	Primary	−	−	45 (100)
	Adjuvant	145 (21.2)	−	−
RCT	Primary	−	40 (100)	−
	Adjuvant	22 (3.2)		−

^a^Mean (Std.Dev)

**Fig. 1:**
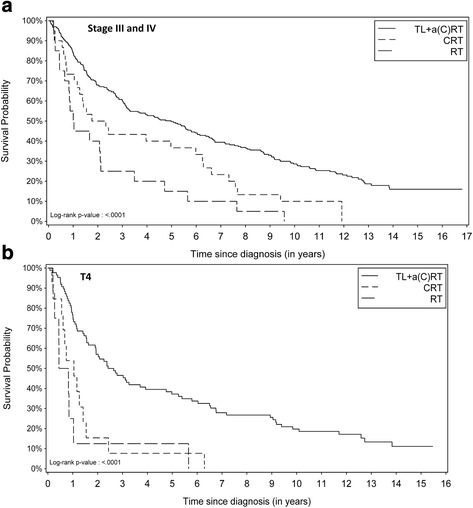
**a** Kaplan Meier curves of stage III and stage IV patients by therapy group (OS); **b** Kaplan Meier curve for T4 carcinoma patients by therapy group (OS)

Our evaluation revealed that when the stage III and stage IV patients were considered together, the patients who received CRT had a non-significantly worse outcome in terms of OS than those who underwent upfront TL (Fig. 1a). The corresponding multivariate Cox proportional hazard analysis showed a difference in OS between the RT and the surgery group (HR 1.92; 95% CI: 1.16–3.19; *p* = 0.0117) but no significant difference in survival between the CRT and the immediate surgery group (HR 1.23; 95% CI: 0.82–1.86; *p* = 0.31).

However, the Kaplan Meier curve for the subgroup of T4 carcinoma patients (*N* = 107; 13.9%) revealed severely compromised survival after conservative LP (log-rank test: *p*-value < 0.0001, Fig. 1b). This was confirmed with the multivariate Cox proportional hazard analysis: Not only was OS worse after RT compared with the immediate surgery group (HR 4.6; 95% CI: 2.1–9.8; *p* = 0.0001), but more importantly, survival was also worse after CRT (HR 2.0; 95% CI: 1.04–3.7; *p* = 0.0369) (Table 2). Approximately 90% of the T4 patients died within 1 year after RT and within 2.5 years after CRT. Not a single T4 patient survived 7 years after primary conservative therapy, whereas the 10-year OS was 20% after TL + aR(C)T (95% CI: 9%–28%).

**Table 2:** Univariate and multivariate Cox proportional hazard analysis results for all T4 patients (*N* = 107), 1998–2015

Characteristic	Category	Deceased	Survived	HR (crude)^a,b^	95%-CI (crude)^a,b^	*p*-value^b^	HR (adjusted)^a,c^	95%-CI (adjusted)^a,c^	*p*-value^c^
Therapy	TL + a(C)RT	74 (77.9)	12 (100)	1	-	-	1	-	-
	CRT	13 (13.7)	0 (0.0)	3.0	(1.6, 5.6)	0.0004	2.0	(1.04, 3.7)	0.0369
	RT	8 (8.4)	0 (0.0)	4.2	(2.0, 8.9)	0.0002	4.6	(2.1, 9.8)	0.0001
Age^d^	(10 year units)			1.3	(1.1, 1.6)	0.0085	1.4	(1.1, 1.7)	0.0014
Recurrences	No	74 (77.9)	12 (100)	1	-	-	1	-	-
	Yes	21 (22.1)	0 (0.0)	8.5	(5.1, 14.7)	<.0001	7.3	(4.1, 12.9)	<.0001
N-stage	N0,N1	56 (58.9)	10 (83.3)	1	-	-	1	-	-
	N2,N3	39 (41.1)	2 (16.7)	2.2	(1.5, 3.4)	0.0002	1.6	(1.0, 2.5)	0.0489
Tumour location	glottic	18 (18.9)	0 (0.0)	1	-	-			
	supraglottic	34 (35.8)	4 (33.3)	0.75	(0.42, 1.3)	0.3282			
	subglottic	6 (6.3)	2 (16.7)	0.62	(0.24, 1.6)	0.3067			
	transglottic	26 (27.4)	3 (25.0)	0.74	(0.40, 1.4)	0.3300			
	Unknown	11 (11.6)	3 (25.0)	0.71	(0.33, 1.5)	0.3706			
CCI^e^	None	56 (58.9)	11 (91.7)	1	-	-			
	One and more	39 (41.1)	1 (8.3)	1.8	(1.2, 2.7)	0.0061			
2nd primary carcinoma	None	88 (92.6)	11 (91.7)	1	-	-			
	Yes	7 (7.4)	1 (8.3)	1.3	(0.60, 2.9)	0.4857			

^a^HR: Hazard Ratio; CI: Confidence interval; ^b^Results from univariate analysis; ^c^Results from multivariate analysis using backward selection; ^d^continuous, ^e^CCI: Charlson Comorbidity Index

In the 179 references cited as evidence in the ASCO guidelines, five classical LP studies were found. Four of these five studies included T4 cancer patients. Differential outcome data on treated T4 tumor patients were presented in three of these four studies. In one of these three studies, the number of patients who did not respond to induction chemotherapy was not given. These patients were part of the conservative treatment arm but received upfront TL + adjuvant radiotherapy. Thus, the exact number of T4 patients in the conservative treatment arm of that study who eventually received conservative treatment was unclear. Thus, differential outcome data were presented for only 21–45 T4 tumor patients. These data, however, show a markedly worse outcome for the T4 subgroup (Table 3).

**Table 3:** Summary of patient outcomes in 5 studies comparing LP and TL in advanced laryngeal tumors

Study	T4 patients assigned to conservative treatment arm	T4 patients eventually treated by primary CRT or RT	Comments
VALCSG [4, 14]	43	Unclear, 19 < *N* < 43	59 TL in 116 T1-T4 patients in the conservative treatment arm, 30 upfront 24 TL (upfront + salvage) in 43 T4 patients TL in T4: 56% TL in T1–3: 29% T4 had 5.6-fold lower probability to achieve response to ICT T4 had 7.1-fold poorer organ preservation rate than T1–3
EORTC [1]	4	0	No T4 patient in the chemo arm eventually received conservative treatment, i.e. upfront TL followed by RT was the treatment for all T4 patients in the surgery as well as in the chemo arm
GETTEC [15]	0	0	Only T3 patients were included TL in 58% of patients of ICT arm OS after CRT significantly poorer than after surgery (*p* = 0.006)
Bhalavat [16]	2	2	1 local recurrence after partial remission 1 survived for 5 years
RTOG 91–11 [2, 8, 17, 18]	18 ICRT 17 CCRT 16 RT	Unclear	No T4 tumor with penetration through the cartilage, cartilage at the most minimally eroded 7 upfront TL in ICRT No data given about T category No differential data given for T4

## Discussion

In the observational study, survival among T4 patients was significantly worse when their larynx was not removed as part of the primary treatment regimen. This result contrasts with the 2006 ASCO clinical guidelines’ statement that LP methods result in equal survival compared with primary surgery. Although the number of T4 patients in the CRT and RT groups was small, the data present the outcome of a representative cohort of all laryngeal cancer patients within a population of 2.7 million inhabitants.

Hospital records were used to extract data on disease-specific characteristics, socio-demographic variables of the study population and any events after diagnosis. The presence of comorbidities in 28.6% of the patients is likely to be an underestimation as information about comorbidities might be collected differently by physicians in different hospitals. Although validity could not be verified, the comorbidities recorded at the time of diagnosis should present a non-differential bias at the most and therefore should not have led to an overestimation of the real effect or interfered with the other variables in our analysis.

The Veterans Affairs Laryngeal Cancer Study Group (VALCSG) and the European Organization for Research and Treatment of Cancer (EORTC) trials proved that LP with induction chemotherapy followed by radiotherapy (ICRT) was feasible for advanced laryngeal and hypopharyngeal cancer patients without jeopardizing survival [1, 2]. However, the question is whether these large, randomized trials yielding level I evidence [9] provide sufficient evidence that LP is as appropriate for early T4 patients as for T3 patients, as stated in the 2006 ASCO guidelines. In the EORTC hypopharyngeal trial, [2] induction chemotherapy (ICT) served as stratifier for patients who might profit from mere conservative treatment. Not a single T4 disease patient responded to ICT with complete remission. Thus, no T4 patient in this study received primary RT, but all of them were treated with upfront TL and aRT. The VALCSG laryngeal cancer study [1] is the largest prospective randomized controlled trial to date of laryngeal cancer patients; it included 332 stage III and IV patients, with 42 and 43 T4 patients in the two treatment arms. In total, 59 of the 116 patients in the conservative arm underwent TL: 30 before and 29 after RT. “Salvage laryngectomy was required, however (…) in 56 percent of the patients with T4 cancers compared with 29% of patients with smaller primary tumors (p=0.0001).” [1]. Further multivariable analysis in 1999 revealed that T4 tumors had a 5.6-fold lower likelihood of responding to chemotherapy than T1–3 tumors (95% CI, 1.5–20.8; *p* = 0.0108) [14]. The full multivariate model for predicting LP in patients treated with ICRT showed that T4 patients had a 7.1-fold worse organ preservation rate than T1–3 patients (95% CI, 1.7–29.5; *p* = 0.0070) [14]. In other words, T4 tumor patients had a markedly higher risk of failure after ICRT.

The Groupe d’Etude des Tumeurs de la Tête et du Cou (GETTEC) study [15] included only T3 laryngeal carcinoma patients. Although these patients’ tumors were less advanced than T4, 21 of the 36 patients in the ICT group were treated with TL (58%), and despite salvage TL, “survival and disease-free survival were significantly worse in the induction chemotherapy group than in the no chemotherapy group (*p*=0.006 and *p*=0.02, respectively)” [15]. Richard concluded that “larynx preservation for patients selected on the basis of having responded to ICT cannot be considered a standard treatment at the present time.” [15] Consistently, the GETTEC study was stopped because of these poor results for patients with fixed cord cancer [16]. Although fixation of the vocal cord does not surpass the T3 criteria, Horn interpreted the poor results as a logical consequence of tumors with a worse prognosis per se [6]. These results suggest that LP might reach its limits of efficacy even in less advanced stages than T4.

In the Bhalavat study [17], there were only two patients with a T4 tumor in the radiotherapy arm compared with seven T4 patients in the primary surgery arm. One of the two patients treated with RT relapsed after a moderate response, while the other one survived for 5 years. In contrast, the T4 patients treated with primary TL had a 5-year OS of 75%. However, it is impossible to draw any reliable conclusions from these results.

The Intergroup RTOG 91–11 study was supposed to show the non-inferiority of concomitant chemo-radiotherapy (CCRT) compared with the VALCSG induction chemotherapy regimen (ICRT) [3]. Provided that the OS outcome after ICRT was superior to that after CCRT, non-responsiveness to induction chemotherapy might identify the patients who require a more radical treatment strategy than primary CRT alone (as was the case for all the T4 patients in the EORTC study). The non-responders in the ICRT arm received primary TL followed by RT and thus were likely to have outcomes comparable to those of the patients in the surgical arm of the VALCSG study; however, this stratification was missing in the CCRT arm. In the RTOG study, CCRT was superior to ICRT and RT alone in terms of larynx preservation, and the five-year OS estimates did not differ significantly. However, the recently published long-term results showed 10-year OS rates of 38.8% and 27.5% in the ICRT and the CCRT groups, respectively. Although it was not statistically significant (*p* = 0.08; HR 1.25; 95% CI 0.98–1.61) [18], this strong effect cannot be ignored [8]. The RTOG study cohort contained a mix of approximately 10% T2 patients, 30% T3 patients without cord involvement, 50% T3 patients with fixed cord involvement, and only 10% T4 patients in each group. Nonetheless, earlier-stage tumors have a much better responsiveness to chemo-RT. Thus, a marked statistically significant difference could be anticipated if a T4 subgroup analysis were performed. However, a statistical comparison among the T4 patients in the three treatment arms was precluded, according to Forastière, as “only 10% of patients enrolled in RTOG 91-11 had T4 cancers” [19]. There were 18, 17, and 16 T4 tumor patients in each arm of the study and a huge number of other stage III and IV patients with T2 and T3 tumors. Desirably, within the same treatment arm, the outcome of this relatively small number of T4 patients could be compared with those of the large number of T2 and T3 patients. This subgroup analysis might provide revealing level I evidence of the outcome of T4 laryngeal cancer patients compared with lower T stage patients after different types of LP.

In the RTOG 91–11 trial, the reported successful salvage TL rates after CCRT and ICRT were 69% and 71%, respectively [20]. The salvage TL success rate, however, depends on the T category. Johansen reported a salvage TL success rate of 79% for T1a, 68% for T2, 60% for T3 and only 44% for T4 glottic carcinoma [21]. Parsons reported a success rate of 25% for T4 tumors compared with 50% successful salvage TL for other T categories [22]. Thus, the salvage TL success rate for T4 larynx carcinoma is not as favorable as the overall success rate of approximately 70% reported for all T categories in the RTOG 91–11 trial; instead, it is 25–50%.

In summary, the RTOG 91–11 does not prove the non-inferiority of CCRT compared with ICRT in T4 larynx carcinoma in the absence of differential data for T4 patients. In the long run, the survival outcome after CCRT was increasingly worse than after ICRT. After 10 years, the difference reached almost statistical significance for the whole treatment arm, which comprised T2, T3, and T4 tumors. This effect is probably more pronounced in the subgroup of T4 tumor patients, who were less responsive to CRT and had a worse outcome with salvage surgery. Forastière stated in 2015 that in her study, “no level I evidence supports a non-operative organ preservation strategy for patients with T4a disease and penetration through cartilage”. These patients were not eligible for the RTOG 91–11 study; “only patients with minimal cartilage erosion” were included [7], and mere cartilage erosion is a notable criterion for a T3 disease in laryngeal cancer. In the other LP studies cited in the ASCO guideline, a total of 21 to 45 T4 patients eventually received primary conservative LP treatment (see Table 2). Thus, the grade I evidence for LP in T4 in these studies is based on a rather low number of patients. Additionally, in terms of differential results, the T4 patients showed a markedly worse outcome after LP compared with the other stage III and IV patients.

Shortly after the establishment of the ASCO guidelines in 2006, some studies were published that supported our finding that a conservative LP approach compromises survival in T4 laryngeal cancer patients. Chen [23] evaluated the outcome of 10,590 patients with advanced laryngeal cancer registered in a national hospital-based cancer registry. Over 900 T4 tumor patients were treated using a primary conservative approach (CRT, *n* = 358; RT, *n* = 566), and 1690 patients were treated with upfront TL + aRT. Among patients with stage IV disease, TL was associated with significantly greater survival than CRT or RT (*p* < 0.001) [23]. “Because the choice between chemo-RT and TL as optimal treatment for patients with T3 primary cancers is a matter of debate” [23], Chen performed a separate proportional hazards (PH) analysis for patients with T3 primary laryngeal cancers. T3 patients treated with CRT had a significantly increased risk of death compared with those treated with TL (HR = 1.18; *p* = 0.03). The effect was even more pronounced for those treated with RT (HR = 1.59; *p* = 0.001). Separate analyses of T4 patients were not performed. In a large monocentric retrospective case series of 451 patients, Gourin collected 50 primarily non-surgically treated T4 patients compared with 77 surgically treated T4 patients over 17 years [24]. After controlling for nodal status, the authors found an increased HR of death for patients treated with CRT (HR 2.0) or RT (HR 7.2) compared with TL + aRT. The 5-year OS of these T4 tumor patients was significantly better after TL + aRT (55%) than after CRT (25%) or RT (0%; *p* < 0.0001) [24].

Accordingly, Olsen pronounced severe concern regarding the actual treatment of T4 laryngeal carcinoma [16]. He especially stated that the distinction of “low-volume T4 tumors from T4 tumors” based on examination or imaging “has not worked and is unproved” [16]. “Tumors that extend through the laryngeal cartilage should be treated with total laryngectomy” [16].

Five recent database studies corroborate the finding of a significant loss of survival after LP in T4 patients. Grover investigated the outcome of 969 T4a laryngeal cancer patients, most (64%) of whom were treated with LP-CRT [11]. He reported a markedly worse outcome for patients treated with LP-CRT compared with patients treated with upfront TL. “Median survival for TL versus LP was 61 versus 39 months (p<0.001)” [11]. CRT showed an inferior OS compared with TL (HR, 1.31; 95% CI 1.10–1.57) after potential confounders were controlled [11]. Megwalu reported 5394 advanced-stage laryngeal carcinoma that were treated between 1992 and 2009. During this period, the rate of non-surgical treatment increased from 32% to 62%. The subgroup of T4 N0 patients who received surgical treatment had a better 5-year OS (56% vs. 38%; *p* < 0.001) than patients who underwent non-surgical treatment, and this effect was markedly more pronounced than that for T3 N0 patients (59% vs. 48%; *p* < 0.001) [25]. In multivariable analysis controlling for potential confounders, non-surgical patients had worse OS (HR, 1.32; 95% CI, 1.22–1.43) than surgically treated patients.

Evaluating 258 laryngeal cancer patients in a prospective longitudinal population-based cohort study, Dziegielewski reported 5-year OS rates for T4a cancers of 70% for TL + a(C)RT, 52% for CRT and 18% for RT. [26] The HRs for RT and CRT compared with TL + a(C)RT were 4.9 (*p* < 0.001) and 2.3 (*p* = 0.04), respectively. It is worth noting that in terms of tumor site, the patients were “balanced with nearly a 50/50 glottic/supraglottic split”, while in the VA and RTOG trials, there was a heavy bias toward supraglottic tumors, which are well known to respond better to CRT. Furthermore, patients with increasingly advanced disease were treated with TL + a(C)RT. Nevertheless, the surgically treated patients had a much better outcome. Moreover, Dziegielewski called attention to the fact that the pivotal LP trials were performed when the AJCC (5th edition until 2002) classified minor cartilage invasion tumors as T4 lesions. “These patients would be downstaged to T3 lesions by today’s standard” [26]. The exclusion of patients with a low Karnofsky index, the inclusion of more supraglottic tumors, and the consequent restriction to T4 tumors, e.g., those with “minimal thyroid cartilage invasion or suspicion of invasion on imaging” per protocol in RCTs constitute “selection bias” [27]. Sanabria critically states that the results of the randomized controlled LP studies are more favorable than those of observational cohort studies and may not generally be extrapolated to standard practice [27].

Timmermans reported the outcome of 1722 T4 laryngeal cancer patients treated in The Netherlands between 1991 and 2010 [28]. The difference in survival outcome compared with the other three recent population-based studies [11, 25, 26] was less marked but was statistically significant: The 5-year OS after TL + a(C)RT, CRT and RT was 48%, 42%, and 34%, respectively (overall *p* < 0.0001) [28]. It is worth noting that the cohort comprised a considerable number of tumors that would be classified as T3 according to today’s standard.

In a long-term retrospective analysis of 221 T4 patients (TL + a(C)RT; *n* = 161, CRT; *n* = 51, and RT; *n* = 9), Rosenthal reported an initially superior locoregional control with upfront TL compared with LP (log-rank *p* < 0.007) [29]. However, successful salvage surgery resulted in an equal median survival time of 64 months in the TL+(C)RT group as well as the LP groups. However, the preponderance of nodal positivity in the surgery group must be taken in consideration (35.5% N2/3 in the TL + aR(C) group vs. 11.5% in the LP group) as the study revealed that node positivity represented the “primary determinant of mortality” (*p* < 0.0001) [29].

A recent database analysis presented the results of 3682 T4 M0 laryngeal cancer patients diagnosed from 2004 through 2012 [30]. Stokes divided the LP cohort into ICRT and CCRT groups (TL + a(C)RT, *n* = 1599 compared with CCRT, *n* = 1597, and ICRT, *n* = 386). The comparison between TL + a(C)RT and CCRT strongly confirms the superiority of surgery over conservative LP in T4 patients in terms of OS (HR, 1.55; 95% CI 1.41–1.70, *p* < 0.01). The ICRT cohort was defined as “undergoing RT plus multi-agent chemotherapy with chemotherapy starting 43 to 98 days before RT” [30]. According to this definition, non-responders to IC and patients discontinuing therapy due to death or no lethal toxicity during the induction period were not included in the ICRT cohort. As it is difficult to identify intention-to-treat ICRT patients in a database analysis, there is no evidence to date that ICRT might yield OS results comparable to those of TL + a(C)RT.

In a systematic review, Francis retrieved 24 retrospective studies reporting survival outcomes in T4 laryngeal cancer patients. The 5-year OS outcome ranged from 10% to 80.9% for surgery, 16% to 50.4% for CRT, and 0% to 75% for RT. However, due to major heterogeneity among the studies in terms of inclusion/exclusion criteria, laryngeal subsite, neck staging and treatment protocols, no clear conclusions can be drawn from these trials [31].

A multidisciplinary international consensus panel developed recommendations for conducting phase III clinical trials of LP in patients with locally advanced laryngeal and hypopharyngeal cancer. The panel explicitly considered whether patients with T4 disease should be eligible for future organ preservation trials “because these patients may suffer worse outcomes with this approach” [32]. This statement was supported by substantial literature. According to the consensus panel, the inclusion criteria for further LP studies are “T2 or T3 laryngeal or hypopharyngeal SCC not considered for partial laryngectomy” but not T4 carcinoma [32]. However, the NCCN treatment guidelines state that while the first recommendation for T4a tumor patients is laryngectomy, concurrent chemoradiation should be considered for “selected T4a patients who decline surgery” [10]. This recommendation is difficult for two reasons: 1.) Almost every patient will naturally reject laryngectomy if offered possible organ preservation as an alternative, especially when preservation is mentioned in current guidelines. 2.) The term “selected” implies that there might be T4a tumors for which a conservative, larynx-preserving treatment might be an appropriate approach. Forastière claimed as recently as 2015 that “selected low-volume T4 tumors endorse concomitant cisplatin and RT on the basis of level I randomized controlled trial data” [7]. As evidence, she quotes the 2006 ASCO clinical practice guidelines for LP [4]. However, our reevaluation of the differential data from the T4 laryngeal cancer patients in precisely these cited studies shows a strong indication that this subgroup has a significantly worse outcome when treated non-surgically. A meta-analysis of the updated data of all T4 patients treated with primary conservative LP in the cited studies compared with the T2 and T3 patients in the same treatment arms could further substantiate this finding.

In summary, the evaluation of the differential data published in the large randomized controlled LP trials for the subgroup of T4 tumor patients revealed that CRT compromises survival in T4 laryngeal cancer patients. Until now, this effect was blurred by the evaluation of all stage III and IV patients together in a group that comprised T2, T3, and T4 patients. Recent large retrospective database studies, a large series with contemporaneous controls, and our own observational cohort study have shown a statistically significant loss of survival in T4 patients treated with conservative LP.

## Conclusions

CRT and RT should no longer be recommended as equivalent treatment options for T4 laryngeal cancer patients, even in selected cases. T4 tumor patients who definitively reject laryngectomy should be informed that the possibility of larynx preservation with primary conservative treatment will likely result in a significantly worse outcome in terms of overall survival. A statement to this effect should be added to the NCCN guidelines.

## Abbreviations

ASCO: American Society of Clinical Oncology
CCI: Charlson comorbidity index
CCRT: Concurrent chemo-radiotherapy
CI: Confidence interval
CRT: Primary chemo-radiotherapy
EORTC: European Organization for Research and Treatment of Cancer
GETTEC: Groupe d’Etude des Tumeurs de la Tête et du Cou
HR: Hazard ratio
ICRT: Induction chemo-radiotherapy
ICT: Induction chemotherapy
LP: Larynx preservation
NCCN: National Comprehensive Cancer Network
OP+/−a(C)RT: Radical surgery with or without adjuvant radiotherapy or adjuvant chemo-radiotherapy, as indicated by stage
OS: Overall survival
PH: Proportional hazards
RCT: Randomized controlled trial
RT: Primary radiotherapy alone
SPC: Second primary carcinoma
TL + a(C)RT: Upfront total laryngectomy followed by adjuvant radiotherapy or chemo-radiotherapy
TL: Total laryngectomy
VALCSG: Veterans Affairs Laryngeal Cancer Study Group
